# Dentitions of Long-Term Care Residents: Tooth Types, Roles in Occlusion and Association with Dementia

**DOI:** 10.3390/healthcare12181886

**Published:** 2024-09-20

**Authors:** Jesse Tervonen, Lina Julkunen, Riitta K. T. Saarela, Kaija Hiltunen, Päivi Mäntylä

**Affiliations:** 1 Institute of Dentistry, University of Eastern Finland, 70211 Kuopio, Finland; jesse.tervonen@gmail.com; 2 Oral and Maxillofacial Diseases Outpatient Clinic, Helsinki University Hospital, 00290 Helsinki, Finland; lina.julkunen@fimnet.fi; 3 Social Services, Health Care and Rescue Services Division, Oral Health Care, City of Helsinki, 00530 Helsinki, Finland; riitta.saarela@hel.fi; 4 Department of Oral and Maxillofacial Diseases, University of Helsinki, 00014 Helsinki, Finland; kaija.hiltunen@helsinki.fi; 5 Oral and Maxillofacial Diseases, Kuopio University Hospital, 70210 Kuopio, Finland

**Keywords:** tooth type, occlusion, dementia, long-term care, caries, periodontitis

## Abstract

**Background/Objectives:** Many older adults living in long-term care (LTC) environments have varying numbers of retained natural teeth. The objective of this study was to assess the disease findings based on tooth type and estimate the role of tooth types in occlusion. **Methods:** We conducted clinical oral examinations of 276 LTC residents. The disease findings were analyzed for each tooth type and to determine their associations with dementia. **Results:** In total, 67.8% of the participants had molar teeth. Premolars/canines were often present as residual roots and had caries. Deepened periodontal pockets and higher plaque index (PI) values for molars had odds ratios of 2.5 (95% confidence interval [CI] of 1.59–3.91) and 1.61 (95% CI of 1.29–2.02), respectively. Participants with dementia were more likely to have incisors and premolars/canines in the form of root remnants and higher PI scores for all tooth types (*p* ≤ 0.01), as well as more deepened periodontal pockets in all teeth (*p* = 0.029), than those without dementia. The median number of remaining molars per participant was 3 out of a maximum of 12 (95% CI 3.4–4.0); thus, they often lacked occlusal contact. **Conclusions:** The LTC residents’ molars were more prone to periodontal problems, whereas their premolars/canines often had caries or were present in the form of root remnants, especially in the participants with dementia. People’s teeth should be treated in an easy-to-maintain way before they move into LTC to enable daily oral hygiene measures and maintain oral health.

## 1. Introduction

Edentulousness has decreased among aged people in recent years [1,2], and older adults in long-term care (LTC) have varying numbers of preserved natural teeth [1,2]. In 2009, it was estimated that 75% of baby boomers will enter LTC as dentate, and this trend is expected to continue in the future [3]. Studies show that LTC residents often have poor oral health, and they are prone to oral diseases [1]. The most common oral health problems in these individuals are plaque-induced, such as periodontal diseases and dental caries [4]. The effect of age itself on the morbidity of caries and periodontitis is complex. Age can directly affect individuals’ morbidity by altering their immune defense, cellular senescence and/or wound healing [2]. Indirect effects can impact individuals by decreasing their physical and cognitive functions, which often leads to difficulty in maintaining oral health [2,5].

A recent review concluded that many older adults living in LTC have active and untreated coronal and root carious lesions, although these conditions are not present in all dentate residents. The data on periodontal conditions are less clear, but it has been estimated that one in five older adults in LTC might benefit from intensive periodontal treatments [6]. The possible underlying reasons for this fact are that oral health is not highly prioritized by staff in residential care facilities and that older adults living in LTC have difficulty accessing oral healthcare [7,8]. These factors, together with the fact that LTC residents’ own ability to maintain their oral hygiene is often inadequate, may further deteriorate their oral health. [4,9]. In such populations, poor oral health and increased oral disease burden are associated with poor functioning and cognitive impairment [7,10,11,12]. 

People with dementia have been identified as an LTC resident subgroup with a particularly high risk of poor oral health [13]. Their natural teeth being in poor condition causes an additional burden in old adults with sensory deficiencies, functional impairments and cognitive decline and presents a considerable risk for fragility in old age [14]. Increasing evidence shows that Alzheimer’s disease may be associated with oral inflammatory diseases through the invasion of oral pathogens into the brain [15,16,17]. Cognitive decline has been associated with changes in the composition of the oral microbiota, although oral health and declining cognition may have a bi-directional association [18].

More than two-thirds of older adults in LTC are missing more than 10 teeth, and, thus they lack a functional dentition [6]. Tooth loss can be considered to be a long-term indicator of oral health [19]. In older adult populations, poor oral health is indicative of earlier-life experience of oral diseases and individuals’ behavioral habits, including poor daily oral hygiene, as well as oral health professional’s failure to provide preventive and professional dental care [19]. 

In general, the upper molars are the most frequently lost teeth in older patients who have medical, physical or cognitive impairments and whose oral health and healthcare are compromised by financial or accessibility factors [20]. Nevertheless, LTC residents may have natural teeth with periodontal and cariological problems, which are not functional parts of occlusion, i.e., they have no counterpart, either in the form of a natural tooth or a prosthetic construction [21,22]. 

Treatment planning for older adults with special needs is a challenging task for dentists to perform when the maintainability of the dentition should be the priority. In addition, dentists often also prioritize restorative treatment of the teeth in the oldest population segments, while periodontal health receives less attention [23]. 

In this cross-sectional study, our objective is to assess the disease findings associated with different tooth types among dentate LTC residents living in Helsinki, Finland, and whether dementia is important in this respect. We also aim to evaluate the occlusal role of preserved teeth based on the number of different types of teeth.

## 2. Materials and Methods

The data used in our study were sourced from the Finnish Oral Health Studies in Older Adults (FINORAL) cohort, including 550 older adults living in long-term care (LTC), i.e., nursing homes or assisted living facilities in the City of Helsinki, and who had previously participated in a nutrition study [10]. Individuals who needed prophylactic antibiotics (*n* = 47) and those who had major deficiencies or completely refused (*n* = 35) clinical examination were excluded from this study. A total of 75 individuals died before this study. No other exclusion criteria were used. The total number of participants in this study was 393 individuals. For this cross-sectional study, we included dentate participants (*n* = 276) in the cohort. A participant was considered dentate if he or she had at least one natural tooth or root remnant left. Participation was voluntary, and written consent to participate was given by the participant or by a proxy if the resident concerned was not able to understand the content of this study, such as if they had dementia. The City of Helsinki and the Ethics Committee of the Hospital District of Helsinki and Uusimaa approved the study protocol (HUS/968/2017). This study adhered to the guidelines of the Declaration of Helsinki.

The examinations were conducted between September 2017 and January 2019. The examination consisted of questionnaires carried out by the registered and trained nurses who were most familiar with the participants in each LTC facility, and the clinical oral examination was conducted by two dentists who were specially trained to perform this study. The information requested is shown in Table 1. 

The oral examinations were carried out with a normal set of dental instruments and loupes (Merident Optergo MO Ultralight Flip-up, Merident, Lohja, Finland) equipped with headlamps attached (Merident Optergo DeLight LED, Merident, Lohja, Finland). The participants either lay on a bed or sat on a chair during the examination. The course of the clinical oral examination was previously described in detail by Julkunen et al. [10]. The oral examination included visual examination of the lips and oral mucosa (healthy, not healthy); clinical estimation of oral wetness/dryness (normal salivation/signs of oral dryness/dry mouth (according to the Clinical Oral Dryness Score (CODS) by Osailan et al.)) [24]; counting the number of natural teeth, including root remnants; and determination of the use of removable dentures, of food debris on oral or denture surfaces and plaque accumulation (plaque index (PI): 0, no plaque; 4, whole tooth covered with plaque) and the level of gingival inflammation (gingival index (GI): 0, no inflammation; 3, severe inflammation). For the PI and GI, the highest value for each tooth was registered, and the mean value for the whole dentition was calculated. The number of open crown and root caries lesions was registered. The pocket probing depth (PPD) was measured on 4 tooth surfaces (mid- and distobuccal and mid- and mesiolingual) and registered as the deepest PPD for each tooth (<4 mm, 4–5 mm, ≥6 mm). Moreover, bleeding on probing (BOP, yes/no) and tooth mobility in static mode (yes/no) were registered for each tooth. A radiographical examination was not conducted.

First, we analyzed the participants’ characteristics, general health and oral findings by dividing the study participants into 2 groups: those who had molars (Group 1, *n* = 187) and those who did not have molars (Group 2, *n* = 89). 

Secondly, we examined the data for each tooth as the research unit and divided the teeth into 3 groups: molars (736 molar teeth in 187 participants), premolars/canines (1919 premolars/canines in 269 participants) and incisors (1470 incisors in 254 participants).

### Statistics

The categorical variables are described as frequencies and percentages (%), and the continuous variables are described as means and standard deviations (SDs) with ranges or as medians (95% confidence interval, CI), depending on the distribution of the variable. In pairwise comparisons, the Chi-square test was used as the statistical test for categorical variables, and the Mann–Whitney U-test was used for continuous variables. In multiple-group comparisons, the Kruskal–Wallis test with post hoc comparisons adjusted via Bonferroni correction for multiple tests was used. The association between molar teeth and the teeth-related disease findings was determined via confounder-adjusted binary logistic regression analysis (age and number of teeth were used as the continuous covariates, and sex, dementia, diabetes, medications in daily use and type of housing were used as categorized covariates). Statistical significance for the p-values was set at 0.05 for all tests. The collected data were saved to SPSS statistical software (version 27.0.1), (IBM Corporation., Armonk, NY, USA), which was also used to perform the data analysis.

## 3. Results

### 3.1. Characteristics of the Study Participants

The mean age of the participants was 83 years (SD: 8.5), and 73.6% were female. Of all the participants, 48.6% lived in nursing homes, and 51.4% lived in assisted living facilities. Nearly 80% of the participants had dementia (mild to severe), and 39.2% could move independently. Of the study sample, 67.8% had molar teeth (*n*= 187, Group 1), and 32.2% did not have molar teeth (*n* = 89, Group 2) (Table 2). The participants in Group 2 were older (mean: 85.6; SD: 8.1) than those in Group 1 (81.6; 8.3), were more often unable to move independently, were more often fed a pureed diet, had longer stays in the current facility and had experienced fewer oral healthcare visits in the past year.

The mean number of teeth (root remnants excluded) in Group 1 was 17.1 (range: 0–29), and in Group 2, it was 5.04 (0–16) (Table 3). In Group 2, 45.9% of the individuals used removable dentures compared to 6% in Group 1, and these individuals were more likely to have oral mucosal lesions (*p* = 0.019). Most of the study sample (Group 1: 74%; Group 2: 83.5%) showed at least some signs of oral dryness. Group 1 individuals with molar teeth had an average of 6.6 occlusal pairs of natural teeth, and 31.6% of them had ≥10 occlusal pairs. In Group 2 (individuals without molars), the mean number of occlusal pairs of natural teeth was 1.2, and no individuals had ≥10 occlusal pairs. Those with molars also had significantly more preserved natural teeth of every other tooth type (*p*-value for all <0.001).

### 3.2. Findings by Tooth Type

We investigated the tooth-related findings based on tooth type (Table 4). The total sample consisted of 4125 teeth in 254 participants, of which 736 (26.3%) were molars, 1919 were (38%) premolars/canines and 1470 (35.6%) were incisors. The median number of molars per participant was 3 out of a maximum of 12 (95% confidence interval [CI]: 3.4–4.0), and for both premolars/canines (max. 12; 95% CI: 6.0–7.0) and incisors (max. 8; 95% CI: 5.2–5.8), the median number was 6.

Premolars/canines were often root remnants (compared to molars (*p* = 0.001) and incisors (*p* = 0.036)) and had caries (compared to incisors (*p* = 0.002)). Instead, deepened periodontal pockets (a PPD ≥ 4 mm, compared to incisors (*p* = 0.004)) and higher PI values (compared to premolars/canines (*p* = 0.003) and incisors (*p* = 0.002)) were more common in molars. 

**Figure 1:**
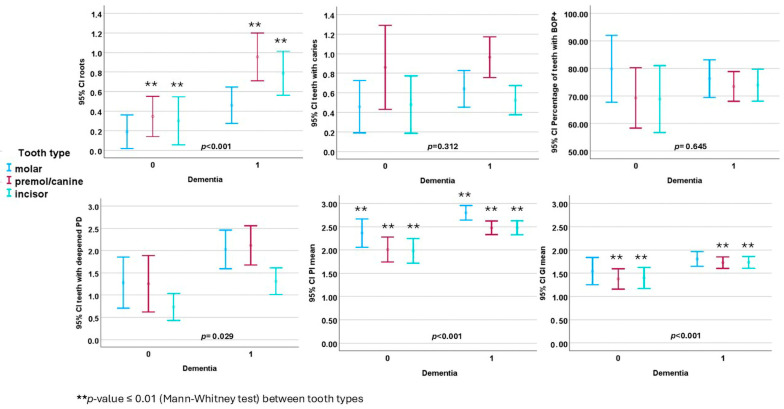
Comparison of tooth type findings between study participants with dementia and those without dementia (mean with 95% confidence interval (CI)).

In the participants with dementia, their premolars/canines and incisors were more often root remnants, and the GI values of both their premolars/canines and incisors, as well as the PI values in all tooth types, were higher than in those without dementia (Figure 1). People with dementia had more teeth with deepened PPDs (*p* = 0.029) compared to those without dementia, with no differences recorded between tooth types.

In a logistic regression model adjusted for age, sex, dementia, diabetes and medications (categorized as ≤5/>5), type of housing and number of teeth, a PPD ≥ 4 mm (OR, 2.5; 95% CI, 1.59–3.91; *p* < 0.001), BOP (OR, 1.01; 95% CI, 1.001–1.012; *p* = 0.026) and the PI (OR, 1.61; 95% CI, 1.29–2.02; *p* < 0.001) were associated with molar teeth. Molars were less at risk of being root remnants compared to the other tooth types (OR, 0.058; 95% CI, 0.36–0.92; *p* < 0.020) (Table 5).

## 4. Discussion

In this cross-sectional study, we assessed the disease findings for different tooth types and estimated their roles in occlusion based on their presence in dentate older adults living in LTC. 

Deepened periodontal pockets and greater bacterial plaque build-up were associated with molar teeth in both the comparison of tooth types and the logistic regression analysis. On the other hand, teeth with dental caries or that were root remnants were more likely to be premolars/canines. Those with dementia typically had higher PI values and deepened periodontal pockets in all their teeth, and their incisors and premolars/canines were more likely to be root remnants compared to in participants without dementia. Participants with molars were more likely to have at least 10 pairs of occluding natural teeth and more preserved natural teeth of each tooth type compared to those without molars. However, in many cases, molars were not in occlusion, judged based on their number and their position in the dental arch.

Oral health problems are an ever-growing challenge among older people around the world [25,26]. Older people are reported to be more susceptible to dental caries and periodontitis [2,5]. Oral health status can have a great impact on health-related quality of life and individuals’ physical and mental health, as teeth enable us to eat and speak, and their esthetic appearance plays a significant part in social contact. Number of teeth is often used as an indicator of good oral health, and the retention of 20+ teeth is found to have a positive impact on the physical functioning of older adults [27]. Hence, teeth-preserving treatment guidelines and retaining teeth healthy are important for maintaining the proper function of the masticatory system. An individual’s care dependency increases when functional and cognitive impairments develop. The treatment choices should therefore favor alternatives through which the dentition can be treated or modified so that it becomes easier to clean and maintain independently or with the help of others [28]. 

It has previously been identified as a limitation of studies conducted in LTC settings that residents with severe diseases may be excluded from the sampling, and as such, reports may underestimate the oral disease burden among this population [6]. To be eligible for assisted living in Finland, one’s physical or cognitive ability to function must always be clearly impaired. All of our study participants were dependent on outside help. We conducted oral examinations in housing units to include those who were also permanently bedridden; only 40% of the participants could move independently, with or without an aid. 

The main focus of the current study was the burden of disease caused by different tooth types and the active role of preserved teeth in occlusion. To ensure a better numerical distribution between the tooth groups, we put premolars and canine teeth into the same group, which could be criticized. Canines have previously been found to be the best-preserved teeth [29,30]. However, for multiple comparisons between tooth types, premolars/canines were more likely to have caries or be root remnants compared to the other tooth types. The average number of caries lesions observed varied between 0.6 and 1 in the different types of teeth, compared to in previous studies, where the incidence of caries varied between 0.8 and 3.7 when estimated based on all teeth [31,32,33]. An earlier study among patients of an outpatient university clinic indicated that molars are more likely to be affected by caries [34]. However, our study population’s characteristics are quite different from those of patients who seek treatment independently. Instead, the periodontal disease findings were especially associated with molars. The mean number of teeth with deepened periodontal pockets varied from 1.3 to 2, which is somewhat less than an earlier finding among another group of LTC residents, who had a mean of 2.5 teeth with a PPD ≥ 4 mm [33]. Among institutionalized older adults, approximately half have been recognized to have a condition (gingivitis, calculus or a periodontal pocket) requiring periodontal treatment [32]. Periodontal inflammation that is already present favors further plaque formation, which agrees with our results, as their molar teeth had a PPD ≥4 mm more often and had significantly higher PI values than the other tooth types, regardless of the fact that assisted oral hygiene was equally common both in the participants with and without molars [34,35,36]. Posterior teeth, especially maxillary, were found to have higher PI values compared to anterior teeth, as also found in previous studies [37,38].

Almost 80% of the participants had dementia when mild dementia was also taken into account. In earlier studies conducted on this cohort, a high oral disease burden was common and associated with cognitive decline [10,39]. In the current study, among those who had dementia, all of their teeth were more often covered with plaque and had deepened periodontal pockets. In addition, those with dementia had more root remnants compared to those without dementia. Significantly more plaque, worse periodontal status as specified by the CPITN index and more retained roots have also previously been reported in older adults with dementia than in those without dementia [13,40,41]. Delwel et al. [40] also demonstrated that dentate participants with dementia had more coronal caries and root caries lesions than dentate participants with mild cognitive impairment. In our sample, caries was not associated with dementia in terms of either the whole dentition or different tooth types. In this same cohort, we previously detected that impaired cognition as measured with the MMSE was accompanied by poor oral health status and the appearance of major taxa of the gut microbiota in the oral cavity, suggesting the association between oral health and declining cognition may be bi-directional [18]. We cannot draw conclusions about the effect of poor oral health on cognition in our study participants. Rather, it is likely that the participants’ poor oral health is a result of their impaired cognition. Based on our findings, we can argue that the dentitions of LTC residents who participated in this study were not easily maintainable. It is also possible that there were several among those with dementia who repeatedly/continuously refused assistance with oral hygiene. 

Incisors were the best-preserved teeth, having a median number of 6 out of a possible 8; however, this number was clearly lower than that in those who did not have molar teeth. The median number of remaining molars per participant was 3 (ranging between 3.4 and 4), which is somewhat in line with an earlier study, where the mean (SD) number of missing molars in LTC residents was 2.5 (1.5) from the maxilla and was 2.7 (1.3) from the mandible [31]. We also counted wisdom teeth because in many cases, they are the only remaining molars in older people [42]. In the current study, of those who had molars, only 6% used removable dentures. Based on this fact, we can estimate that the majority of their molars were without a counterpart and were not functional parts of occlusion, even though approximately 30% of those with molars had ≥10 pairs of occluding natural teeth, compared to the participants with no molars, of whom none had ≥10 occluding pairs. In addition, the participants with molars also had significantly more preserved natural teeth of every other tooth type. A previous study of the same cohort that also included edentate participants showed that 15% had ≥10 functional occlusal units and that some of the LTC residents, although they had natural teeth left, were completely devoid of occlusal units, whether tooth/tooth or tooth/denture [22]. 

A systematic review that discussed oral health status and the need for oral care in an aging population showed concerns about the potential association between tooth loss due to periodontitis and the onset of dementia [43]. This emphasizes the importance of early diagnosis of periodontitis and treating the periodontium in earlier stages of life. Controlling previously untreated periodontitis is too late in the phase of living in LTC. The periodontal disease findings in the molars may have developed or progressed during LTC habitation, but the presence of many molars with no occlusal counterparts also suggests problems with making treatment decisions once a transition to LTC was made. It is challenging for dentists to make treatment decisions, especially decisions to extract teeth, at the stage when oral healthcare in an older adult proceeds to a phase that should focus on teeth maintenance and palliative care [44,45] while simultaneously striving to maintain functional occlusion and oral health-related quality of life (OHRQoL), which is related to the preservation of natural teeth [46,47]. A change in treatment regimen should be considered when an older adult’s ability to function is significantly decreasing, their need for outside help is increasing and accessing treatment is becoming more difficult. Dentists should note that the prevalence of periodontal diseases increases with age, and age can be a poor prognostic factor for already periodontally damaged teeth [48,49]. All of these facts underline the need to enhance geriatric oral health courses in dental undergraduate and postgraduate education [50].

The strength of our study is its relatively large sample of older adults living in LTC. Studies with such a comprehensive clinical oral examination including detailed clinical information about oral health among frail and vulnerable older adults are sparse. We were able to perform clinical diagnostics, which is important for detecting inflammation, which is possibly linked to general health. To the best of our knowledge, this is the first study to compare the itemized disease findings of different tooth types in this kind of population.

The cross-sectional nature of this study must be considered its main limitation, and cause-and-effect relationships cannot be assessed. Radiographic examinations could not be conducted in LTC, and as a result, it is likely that some oral-disease-related changes were undetected. The lack of radiograph imaging is a particularly significant problem for periodontal diagnostics and more accurate caries diagnostics, such as secondary caries associated with fillings. We did not register fillings, which can be regarded as indicators of earlier caries. A periodontal diagnosis could not be conducted based on the European Federation of Periodontology’s 2018 criteria: the oral examinations were conducted in housing units with the participants sitting on a chair or lying in bed, and the study population consisted of old (mean age > 80 years) and mostly cognitively and/or physically impaired LTC residents who had problems keeping their mouths open for long enough to carry out a thorough periodontal examination. Another significant limitation is that we estimated the functional role of the teeth based the preserved amount of each tooth type, which is a very rough method compared to classification that takes into account all aspects of functionality [51]. We did not have an actual control group. Based on these issues, our results are only applicable to people living in assisted living facilities. A more detailed analysis of the occlusion of LTC residents, together with an analysis of the disease burden in different tooth types in an older population whose ability to function is not as impaired, is still required.

## 5. Conclusions

In conclusion, molar teeth are more likely to be covered in plaque and have more periodontal problems than other tooth types in older adults living in LTC, and they often lack an occlusal counterpart. Molar teeth without a counterpart should be viewed critically when a transition to institutional care is required. Maintaining a healthy natural dentition among LTC residents with cognitive and functional impairments is challenging. Oral healthcare for aged people should consider the time perspective in future and implement treatment in such a way that the dentition can easily be maintained when a transition to assisted living becomes inevitable.

## Figures and Tables

**Table 1:** Data collected by a questionnaire and filled out by the LTC unit nurse.

	Unit or Alternatives
Sex	male/female
Age	years
Type of housing	nursing home/assisted living facility
Length of residence in current facility	months
Need for assistance with oral hygiene	independently/with assistance
Moving	moving independently with or without an aid/not moving independently
Diet	normal/soft
Date of last visit to oral healthcare (to a dentist or oral hygienist)	in the past year/over a year ago
Dementia *	yes/no
Diabetes *	yes/no
Number of medications *	categorized as ≤5/>5

* Extracted from medical patient records.

**Table 2:** The characteristics of study participants segmented based on whether they had molar teeth (Group 1) or not (Group 2).

	Group 1 With Molar Teeth, *n* = 187	Group 2 Without Molar Teeth, *n* = 89	All Combined
Sex, female, *n* (%)	140 (74.9)	63 (70.8)	203 (73.6)
Age, years, mean (SD)	81.6 (8.3)	85.9 (8.1)	83.0 (8.5)
Housing, *n* (%) Nursing home Assisted living facility	97 (51.9) 90 (48.1)	37 (41.6) 52 (58.4)	134 (48.6) 142 (51.4)
Diabetes, *n* (%)	35 (19.3)	16 (19.0)	51 (19.2)
Medications, >5, *n* (%)	127 (70.9)	59 (70.2)	186 (70.7)
Dementia, *n* (%)	142 (79.3)	64 (78.0)	206 (78.9)
Independently moving, with or without an aid, *n*(%)	63 (34.4)	42 (49.4)	105 (39.2)
Diet, normal, *n* (%)	99 (56.6)	54 (66.7)	153 (59.8)
Length of residence *, months, mean (SD)	42.3 (32.3)	51.5 (45.6)	45.3 (37.3)
Visit to oral healthcare in past year, *n*(%)	74 (41.1)	27 (32.5)	101 (38.4)
Daily oral hygiene with assistance, *n*(%)	147 (80.8)	60 (74.1)	207 (78.7)

* Length of residence at the current facility.

**Table 3:** Oral findings of study participants with (Group 1) and without (Group 2) molar teeth.

	Group 1 Having Molar Teeth, *n* = 187	Group 2 Not Having Molar Teeth, *n* = 89	*p*-Value **
* *n* of teeth, mean (SD) (range)	17.1 (7.04) (0–29)	5.04 (4.0) (0–16)	
Removable dentures, *n* (%)	11 (6.0)	39 (45.9)	
Occlusal pairs of natural teeth, mean (SD), range	6.6 (4.6), 15–8	1.2 (2.1), 0–8	
Having ≥10 occlusal pairs of natural teeth, *n* (%)	59 (31.6)	0	
* Molars, mean (SD), range	3.6 (2.3), 0–12	0	
* Premolars/canines, mean (SD), range * Incisors, mean (SD), range	7.8 (3.2), 0–12 5.9 (2.5), 0–8	2.8 (2.2), 0–9 2.4 (2.2), 0–8	**<0.001** **<0.001**
**General oral findings**			
Skin and/or lip lesions, *n* (%)	79 (42.7)	35 (40.2)	0.70
Oral mucosal lesions, *n* (%)	28 (15.6)	24 (27.9)	**0.019**
Mouth dryness, *n* (%) Clinically normal salivation Signs of oral dryness Dry mouth	47 (26.0) 88 (48.6) 46 (25.4)	14 (16.5) 47 (55.3) 24 (28.2)	0.228
Food debris on oral or denture surfaces, *n* (%)	105 (57.7)	41 (48.2)	0.15
**Oral indices**			
BOP-positive teeth %, mean (SD)	82.1 (30.7)	80.4 (35.9)	0.92
PI, mean (SD)	2.5 (0.9)	2.6 (1.1)	0.55
GI, mean (SD)	1.7 (0.8)	1.7 (0.9)	0.67

* Root remnants not included; ** The Chi-square test was used for categorized variables, and the Mann–Whitney U test was used for continuous variables. BOP, bleeding on probing; PI, plaque index; GI, gingival index; PPD, probing pocket depth. Significant *p*-values are depicted in bold.

**Table 4:** Findings by tooth type.

	Molars (M) *n* = 736	Premolars/Canines (P) *n* = 1919	Incisors (I) *n* = 1470	*p*-Value * Multiple Comparisons [Post Hoc]
N of participants	187	269	254	
Per participant, med (95% CI)	3.0 (3.4–4.0)	6.0 (6.1–7.0)	6.0 (5.2–5.8)	
	**Mean (95% CI)**			
Root remnants	0.3 (0.2–0.5)	0.73 (0.6–0.9)	0.44 (0.3–0.6)	**<0.001** [M/P < 0.001, P/I 0.036]
Teeth with caries lesion	0.6 (0.5–0.8)	1.0 (0.8–1.2)	0.6 (0.4–0.7)	**0.002** [P/I 0.002]
Teeth with a PPD ≥ 4 mm	1.9 (1.6–2.3)	2.0 (1.7–2.4)	1.3 (1.1–1.6)	**0.004** [M/I 0.004]
PI	2.7 (2.5–2.8)	2.4 (2.3–2.5)	2.4 (2.2–2.5)	**<0.001** [M/P 0.003, M/I 0.002]
GI	1.7 (1.6–1.9)	1.6 (1.5–1.8)	1.6 (1.5–1.8)	0.32
BOP %	80.1 (74.7–85.5)	75.1 (70.7–79.6)	77 (72.2–81.7)	0.074

* The Kruskal–Wallis test with significant values in pairwise comparisons was adjusted by Bonferroni correction for multiple tests; PPD, probing pocket depth; PI, plaque index; GI, gingival index; BOP, bleeding on probing. Significant *p*-values are depicted in bold.

**Table 5:** The association of oral disease findings with molar teeth adjusted for age, sex, dementia, diabetes and medications, type of housing (nursing home or assisted living facility) and number of teeth.

	OR	95% CI	*p*-Value
PPD ≥ 4 mm	2.5	1.59–3.91	**<0.001**
Caries	1.14	0.86–1.17	0.302
Roots	0.58	0.36–0.92	**<0.020**
BOP	1.01	1.001–1.012	**0.026**
PI	1.61	1.29–2.02	**<0.001**
GI	1.20	0.95–1.52	0.128

PPD, probing pocket depth; BOP, bleeding on probing; PI, plaque index; GI, gingival index. Significant *p*-values are depicted in bold.

## Data Availability

The data may be made available by the authors upon reasonable request and with the permission of the City of Helsinki. Please contact Päivi Mäntylä to make data access requests.
